# Distinct Defects in Synaptic Differentiation of Neocortical Neurons in Response to Prenatal Valproate Exposure

**DOI:** 10.1038/srep27400

**Published:** 2016-06-06

**Authors:** Yoko Iijima, Katharina Behr, Takatoshi Iijima, Barbara Biemans, Josef Bischofberger, Peter Scheiffele

**Affiliations:** 1 Biozentrum, University of Basel, Klingelbergstrasse 50-70, 4056 Basel, Switzerland; 2 Department of Biomedicine, Fachbereich für Physiologie, Universität Basel, Pestalozzistr. 20, CH - 4056 Basel, Switzerland; 3 Roche Innovation Center Basel, F. Hoffmann-La Roche Ltd., Grenzacherstrasse 124, CH-4070 Basel, Switzerland

## Abstract

Autism spectrum disorders (ASDs) are a heterogeneous group of neurodevelopmental disorders characterized by impairments in social interactions and stereotyped behaviors. Valproic acid (VPA) is frequently used to treat epilepsy and bipolar disorders. When taken during pregnancy, VPA increases the risk of the unborn child to develop an ASD. In rodents, *in utero* VPA exposure can precipitate behavioral phenotypes related to ASD in the offspring. Therefore, such rodent models may allow for identification of synaptic pathophysiology underlying ASD risk. Here, we systematically probed alterations in synaptic proteins that might contribute to autism-related behavior in the offspring of *in utero* VPA-exposed mice. Moreover, we tested whether direct VPA exposure of cultured neocortical neurons may recapitulate the molecular alterations seen *in vivo*. VPA-exposed neurons in culture exhibit a significant increase in the number of glutamatergic synapses accompanied by a significant decrease in the number of GABAergic synapses. This shift in excitatory/inhibitory balance results in substantially increased spontaneous activity in neuronal networks arising from VPA-exposed neurons. Pharmacological experiments demonstrate that the alterations in GABAergic and glutamatergic synaptic proteins and structures are largely caused by inhibition of histone deacetylases. Therefore, our study highlights an epigenetic mechanism underlying the synaptic pathophysiology in this ASD model.

Valproic acid (VPA) is a drug commonly prescribed for patients with epilepsy and bipolar mood disorder^1^,^2^. However, during pregnancy VPA treatment elevates the risk for autism and other neurodevelopmental disorders in the unborn child^3^. The mechanisms underlying this prenatal action of VPA remain unclear. VPA does cross the placenta. Thus, VPA exposure may act directly on developing neuronal cells. Moreover, VPA may also modify maternal tissues and indirectly impact neurodevelopment in the fetus.

The impact of *in utero* VPA exposure has been explored in rodent models. Administration of VPA to pregnant rats or mice during the third trimester results in molecular, synaptic and behavioral alterations in the offspring. Thus, *in utero* VPA-exposed offspring exhibit altered social communication, auditory evoked events and restricted and repetitive behaviors^4^,^5^,^6^,^7^,^8^,^9^,^10^. Electrophysiological studies suggest that prenatal VPA exposure in rats leads to enhanced long-term potentiation and local hyper-connectivity through increased synaptic *N*-methyl-D-aspartic acid (NMDA) receptor density in neocortical areas^11^,^12^,^13^, increased frequency of mEPSCs in the amygdala^14^, and impaired cortical inhibitory synaptic transmission^15^.

The mechanisms underlying these alterations are still far from being understood. Anti-convulsant actions of VPA in adult patients are thought to induce γ (gamma)-aminobutyric acid (GABA) synthesis^1^,^16^, thereby elevating inhibitory synaptic transmission. In fact, VPA alters expression profiles of numerous genes by inhibiting histone deacetylases (HDACs)^17^. Such an epigenetic modification by VPA affects neuronal differentiation of multipotent adult neural progenitor cells^18^,^19^. Moreover, short-term VPA exposure of cultured neocortical neurons alters mRNA expression of genes encoding subsets of synaptic proteins such as glutamamic acid decarboxylase (GAD), GABA_A_ receptors and brain-derived neurotrophic factor (BDNF)^20^. However, the molecular mechanisms underlying the pathophysiology relevant for ASD remain unclear.

In this study, we systematically investigated the effect of VPA on synaptic properties with biochemical, cell biological and electrophysiological studies using *in vivo* and *in vitro* VPA models. We uncover distinct defects in synaptic differentiation of neocortical neurons. These effects are mimicked by direct inhibition of HDACs in neuronal cells. Ultimately, the epigenetic modification elicited by VPA results in a hyper-excitable network state which might be fundamental for neuronal dys-function in ASD.

## Results

### Distinct alteration in synaptic proteins in VPA-exposed mice

**Figure 1:**
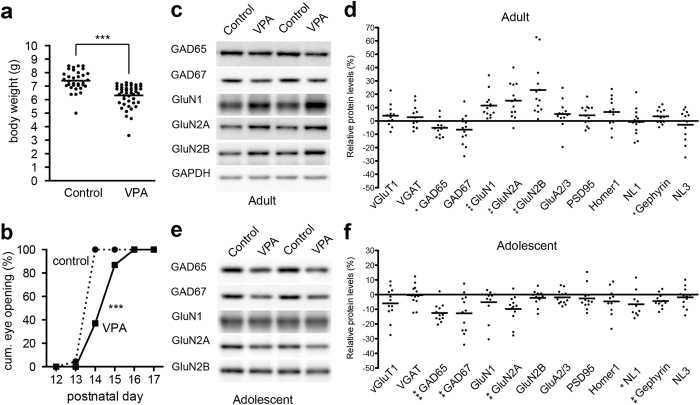
Developmental expression profile of synaptic proteins in *in vivo* VPA model. (**a**) Decreased body weight in the offspring of *in utero* VPA exposed mice at P11 (n = 45 offspring from VPA dams, n = 33 offspring from vehicle treated control animals). (**b**) Developmental delay in eye opening in *in utero* VPA exposed mice (n = 45 offspring from VPA dams, n = 33 offspring from vehicle treated control animals). (**c,d**) Example western blots and quantification of relative expression level of synaptic proteins in somatosensory cortex of adult offspring (postnatal day 70–80) derived from VPA-treated dams (n = 35–43 animals total derived from 12–13 VPA dams, n = 32–40 animals from 15 control dams). Each data point indicates the mean expression level for animals from one dam. The bar indicates the mean of the individual data points across all dams. The % change in expression levels relative to data from vehicle treated control animals is displayed. Blots were cropped to display the relevant molecular weight range. Uncropped blots are presented in Fig. S4a. (**e,f**) Analysis as in (**c,d**) for adolescent VPA-offspring (postnatal day 15–21; n = 42–49 animals from 10–12 VPA dams, n = 44–51 animals from 9 control dams). Blots were cropped to display the relevant molecular weight range. Uncropped blots are presented in Fig. S4b.

To systematically evaluate the effect of *in utero* VPA exposure on synaptic development, we surveyed protein expression of several synaptic and neuronal proteins in large cohorts of offspring from VPA-treated dams (Fig. 1 and Fig. S1). Pregnant mice (outbred CD1 strain) were subjected to subcutaneous injection with VPA (600 mg/kg) or saline at embryonic day 13 (E13). Considering that drug injection and action in each pregnant female represent the biggest experimental variables in such a study we examined cohorts of mice obtained from significant numbers of treated and control dams (n = 13 VPA dams and 15 vehicle dams, >30 offspring per condition analyzed). Dams from both groups had normal pregnancies, and the day of birth was unaffected by VPA exposure, always occurring on E18 or E19. The size of the litters was similar, although there was a tendency for the VPA mothers to have slightly more pups (11.8 ± 0.6 pups/litter in vehicle vs. 14.0 ± 0.4 pups/litter in VPA group, n ≥ 23 litters, mean and SEM, p < 0.01, Mann-Whitney test). Offspring from VPA-treated dams had significantly reduced body weight and exhibited a delay in eye opening (Fig. 1a,b) as well as impaired surface righting and mid-air righting reflexes during the first weeks of life (data not shown).

Protein expression in the somatosensory cortex of adult male and female offspring were examined using quantitative western blots (Fig. 1c–f). Consistent with previous findings^11^, we observed a significant increase in NMDA-type glutamate receptor subunits GluN1, GluN2A and GluN2B in adult offspring from VPA-treated dams (Fig. 1c,d). By contrast, AMPA-type glutamate receptor subunit GluA2/3 was not altered. We then probed the level of several proteins that localize to glutamatergic and/or GABAergic synapses. We observed a small reduction in GAD65 and a trend to decrease for GAD67 (Fig. 1c,d). By contrast, a number of additional synaptic proteins (vGluT1, vGAT, PSD95, Homer1, neuroligin-1) were not altered in adult VPA-offspring. The somatosensory cortex has been implicated in sensory and behavioral alterations associated with autism-spectrum disorders^21^, but many other brain regions are thought to contribute to different aspects of the autism phenotype, including the cerebellum, a center for multisensory integration, fine-tuning, and timing of neuronal responses^22^. Moreover, the volume of hippocampus and amygdala might be reduced in autistic brains^23^. Notably, under our experimental conditions no equivalent alterations in protein levels were observed in the cerebellum, hippocampus and amygdala of VPA-treated offspring. This indicates that the VPA-induced effects on synaptic proteins are brain area-selective (Fig. S1a).

Some aspects of synaptic pathophysiology might be shared between this environmental insult model and genetic models of ASD. Thus, we next investigated expression of GAD65, GAD67, GluN1, GluN2A, GluN2B and gephyrin in the somatosensory cortex of two monogenic ASD models, Fragile X mental retardation protein 1 (FMR1) knockout and Neuroligin-3 R451C (NL3 R451C) knock-in mice^24^. However, we did not observe a comparable alteration in GAD65, GAD67, GluN2B or gephyrin protein levels in either of two genetic models (Fig. S1b). We note that previous studies did report elevation as well as reduction in GAD65/GAD67 protein levels in FMR1 knock-out mice. The divergent findings may be due to difference in age and brain areas analyzed^25^. Interestingly, GluN1 and GluN2A protein levels were increased in the somatosensory cortex of *Nlgn3* R451C mice (Fig. S1b), providing a parallel to the VPA model. In summary, our results support a differential modification of glutamatergic and GABAergic systems in the somatosensory cortex of adult offspring from VPA-treated mice.

ASD is a neurodevelopmental disorder that manifests in early postnatal life. Thus, we next examined whether the molecular alterations identified in adult mice are already present at P15-P21, a developmental time period where cortical circuits undergo significant changes in plasticity and maturation. Notably, we found that the extent and nature of molecular alterations differed between developing and adult somatosensory cortex. The GABAergic synaptic proteins GAD65 and GAD67 were more severely reduced in adolescent mice compared to adult. Thus, reduction in GAD65 was subtle in adult offspring from VPA-treated mice but a more marked reduction in adolescent mice (Fig. 1e,f). Interestingly, the NMDAR subunits which we observed to be significantly elevated in the adult were unchanged or slightly downregulated in adolescent mice. These observations demonstrate that prenatal VPA-exposure results in a down-regulation of GABAergic presynaptic components in the somatosensory cortex of adolescent mice. In adult mice, these GABAergic changes are somewhat attenuated and a significant up-regulation of NMDA-receptors subunits is observed (Fig. 1c,d). When separately analyzing protein alterations in male versus female mice we did not observe significant differences in protein levels. However, some changes appeared to be somewhat more robust in male mice. GAD65 was 5.7% reduced in male offspring but no change (−0.9%) in female offspring. GluN1, GluN2A and GluN2B were 11.7%, 10.7% and 18.8% increased in male, while 9.7%, 6.9% and 10.4% in female, respectively (Fig. S1c). Overall, we noted that the means of protein alterations calculated separately for offspring from each VPA-treated dam showing significant variation (F(11, 46) =3.077 (*P* = 0.0055), one-way ANOVA in GluN2) (Fig. S1d,e). This further supports the notion that for this animal model, individual offspring can not reliably considered as number of experimental replications but rather that average data obtained for each litter should be considered each a single data point.

**Figure 2:**
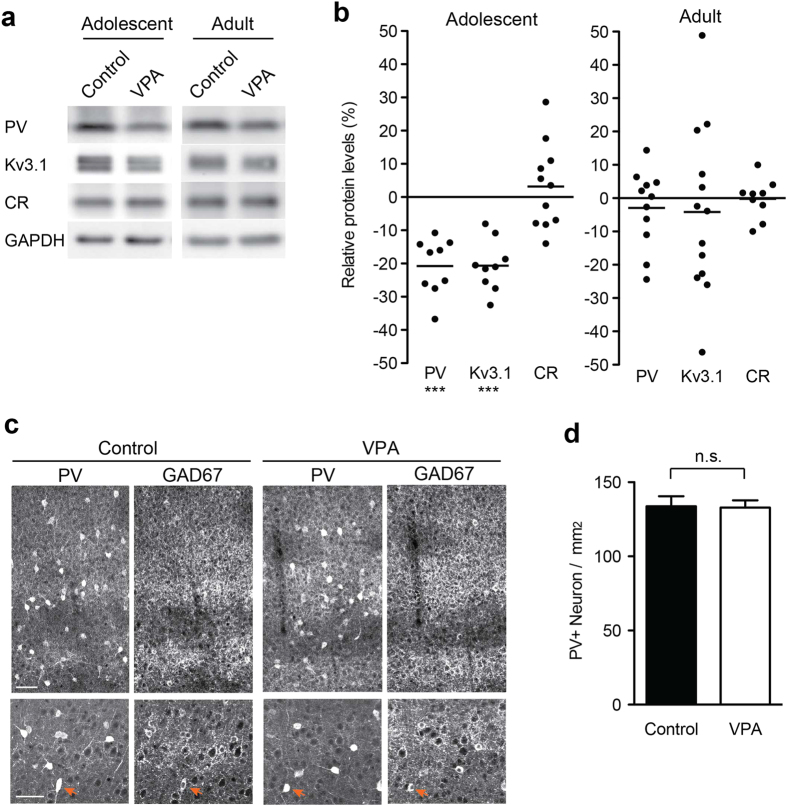
Altered protein level of GABAergic neurons in the adolescent and adult *in vivo* VPA model. (**a,b**) Differential effect of prenatal VPA-exposure on expression of inhibitory neuronal cell-type specific proteins, Parvalbumin (PV) and Calretinin (CR), in adolescent (n = 33–38 animals from 9–11 VPA dams; n = 33–41 animals from 7–9 control dams) and adult mice (n = 29–41 animals from 9–13 VPA dams; n = 30–38 animals from 12–13 control dams). Blots were cropped to display the relevant molecular weight range. Uncropped blots are presented in Fig. S4c. (**c,d**) Number of PV-positive inhibitory interneurons in adolescent mice exposed to VPA *in utero*. Immunohistochemistry on coronal sections from somatosensory cortex were performed with anti-PV and anti-GAD67 antibodies (some double-positive cell somata are highlighted with arrow heads). 32 and 54 confocal images were analyzed from 3 control and 4 VPA animals, respectively. Scale bar = 50 μm.

We further examined expression of inhibitory neuronal cell-type specific markers in the adolescent VPA model. In the neocortex, approximately 70% of GABAergic neurons express either parvalbumin (PV) or calretinin (CR)^26^,^27^. Notably, immunoblot analyses of lysates from adolescent offspring from VPA-treated dams showed a significant reduction in PV (*P* < 0.001; >20% reduced) and Kv3.1, a voltage-gated potassium channel predominantly localized to PV-positive inhibitory neurons^28^ (Fig. 2a,b). These findings represent an interesting parallel to previous reports that the PV-positive area was decreased in somatosensory cortex of FMR1 knockout, NL3 R451C knock-in mice, and VPA-exposed rats^29^,^30^. By contrast expression levels of CR were unchanged. Notably, the number of PV-positive interneurons was not altered in somatosensory cortex of the adolescent VPA model mice (Fig. 2c,d), indicating that there is no significant difference in the specification and survival of PV-positive neurons but rather an alteration in their maturation. Thus, prenatal VPA exposure leads to disturbed maturation of GABAergic interneurons in adolescent mice. These defects persist into adulthood, though in somewhat attenuated form. By contrast, there is an elevation in NMDAR expression and some other glutamatergic synapse components in the somatosensory cortex of adult mice.

### Alteration in presynaptic proteins can be recapitulated *in vitro* by direct treatment of neocortical neurons with VPA

**Figure 3:**
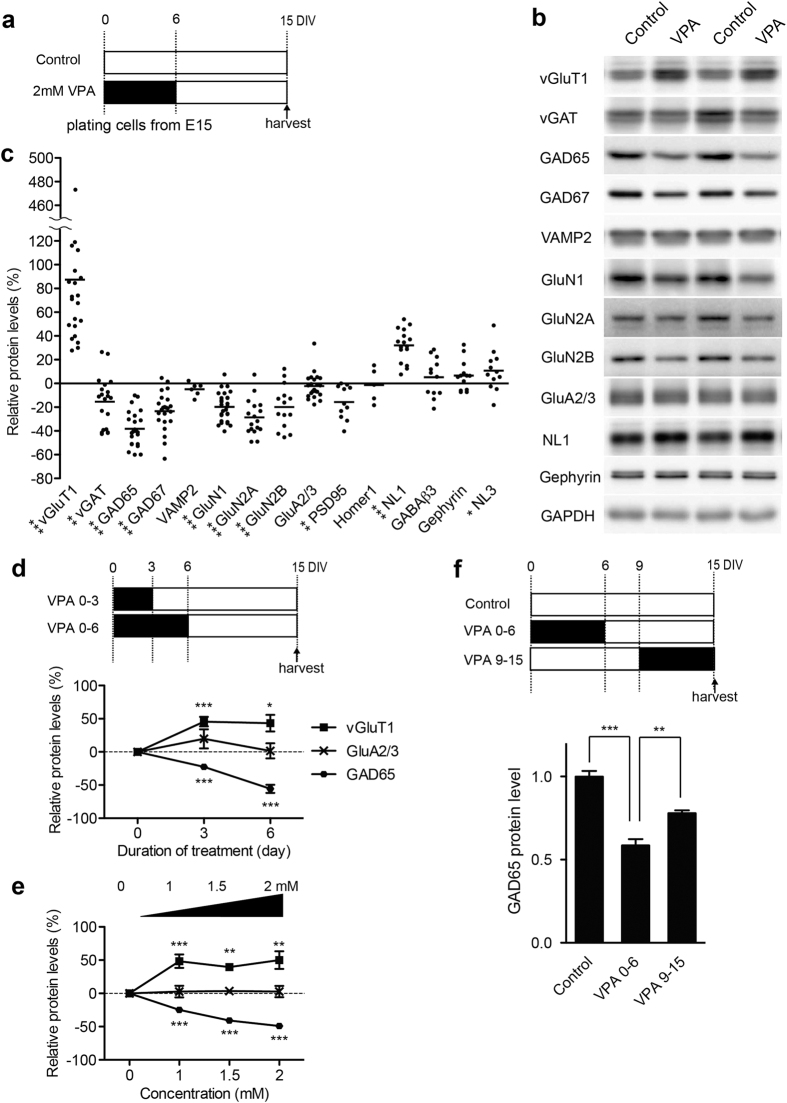
*In vitro* VPA exposure into cultured cortical neurons during early development recapitulates altered expression of presynaptic proteins in adolescent *in vivo* VPA model. (**a**) Scheme for the procedure of *in vitro* VPA exposure. (**b,c**) Example western blots and quantification of relative expression levels of synaptic proteins in cultured neocortical neurons treated with VPA for first 6 days of culture (n = 5–23). The % change in expression levels relative to data from vehicle treated control cultures is displayed. Blots were cropped to display the relevant molecular weight range. Uncropped blots are presented in Fig. S4d. (**d**) Effects of duration of VPA treatment. Neurons were treated with 2 mM VPA for 0, 3 and 6 days. A value in the untreated (0 h) neurons was arbitrarily defined as 0. (n = 3–4). (**e**) Effects of VPA concentrations in cultured medium. Neurons were treated with different concentration of VPA (0, 1, 1.5 and 2 mM) for 6 hrs (n = 3–6). (**f**) Timing-dependent effect of VPA treatment. Neurons were treated with 2 mM VPA for 6 days from DIV0 or DIV9 (n = 4).

Prenatal VPA exposure and other environmental insults may modify neuronal development either directly through action on the developing neurons or indirectly through systemic effects such as inflammatory processes or cytokine signaling. Thus, we tested whether direct exposure of cultured cortical neurons would result in similar synaptic phenotypes as those observed after *in utero* VPA-exposure. Cultured neocortical neurons isolated at embryonic day 15 (E15) from untreated wild-type mice were exposed to 1.0–2.0 mM VPA for 6 days after plating (Fig. 3a). Note, that the effective concentration of VPA in plasma level for the treatment of epilepsy is 50–100 μg/ml (0.3–0.6 mM). The fetal plasma level of VPA is estimated to be 1.3–4.6 times higher than the maternal plasma level^31^. Thus, the foetus is thought to be exposed to a VPA concentration of approximately 0.39–2.76 mM. In the neuronal cultures, VPA-containing medium was completely replaced to normal growth medium at DIV6 (day *in vitro* 6), and cells were maintained until DIV15, a time point when neurons are largely differentiated and matured (Fig. 3a). Notably this *in vitro* VPA exposure had similar effects on the expression of GABAergic markers as observed for adolescent mice that had undergone prenatal VPA exposure. We observed severe reduction in presynaptic proteins GAD65 (38.2%+/−3.1) and slight reduction in GAD67 and vGAT (23.3%+/−3.3, 15.4%+/−4.7 reduced respectively) upon VPA treatment, while the postsynaptic proteins gephyrin and GABARβ3 were not significantly altered (Fig. 3b,c). Moreover, we observed a dramatic increase in the glutamatergic synaptic vesicle marker vGluT1 in the VPA-treated neurons (>1.8 fold increased) (Fig. 3b,c). Interestingly, the effect of VPA on expression of synaptic proteins was dependent on dose, duration and developmental time-point of the treatment (Fig. 3d,e). Thus, a maximum loss of GAD65 protein was achieved at 2 mM concentration and exposure for 6 days. VPA treatment of cultured neurons during the early developing phase (DIV0-6) resulted in a more severe reduction of GAD65 expression (41.5%+/−3.8 reduction) as compared to treatment at a later stage (DIV9–15) (22.0%+/−1.7 reduction) (Fig. 3f). These findings indicate that the dysregulation of synaptic proteins observed after *in utero* VPA-exposure can be replicated by direct VPA treatment of cultured neurons. Moreover, the experiments testing VPA effects in younger *versus* more mature cultures reveal that vulnerability of neurons to the VPA-exposure is particularly high during an early developmental period.

### Disrupted presynaptic differentiation and E/I balance in *in vitro* VPA model

The alterations in presynaptic protein levels resulting from VPA exposure may represent changes in the number, differentiation, and/or function of synaptic terminals or more general changes in the number of viable neurons in the cortical cultures. To distinguish between these possibilities, we morphologically examined neuronal viability, synaptic differentiation, and maturation in VPA-treated neurons using immunocytochemical markers. Immunostaining for MAP2, a neuron-specific marker, showed no obvious morphological abnormalities between untreated- and VPA-treated neurons (Fig. S2a). There was also no significant difference in the number of both CaMKII-positive glutamatergic and GAD67-positive GABAergic neurons (Fig. S2b,c). This is consistent with our observation that the number of PV-positive interneurons *in vivo* is not altered in the offspring of VPA-treated mice (Fig. 2e,f).

**Figure 4:**
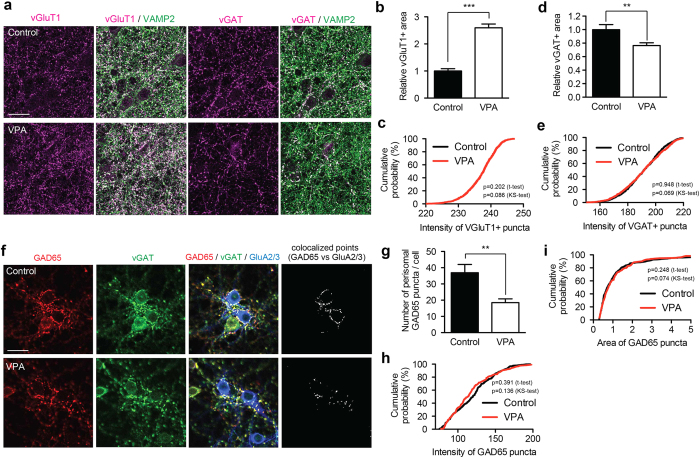
Differential modification of glutamatergic and GABAergic presynaptic terminals in response to VPA in cultured neurons. (**a**) Representative images of vGluT1/VAMP2 or vGAT/VAMP2 protein immunocytochemistry in control or *in vitro* VPA model. The density of vGluT1 positive presynapses was increased and density of vGAT positive presynapses decreased. Scale bar = 20 μm. (**b–e**) Quantification of vGluT1- and vGAT-positive areas and intensity distribution of punta in control and VPA-treated cultures (≥24 images from 3 independent experiments). (**f**) Representative images of GAD65 and vGAT protein immunocytochemistry in control or *in vitro* VPA model (anti-GluA2/3 immunereactivity was used to outline neuronal cell somata). (**g–i**) The number of GAD65 positive perisomatic puncta per cell was reduced in VPA treated cultures, whereas intensity and size of GAD65 puncta were not altered. Scale bar =20 μm (≥15 images from 3 independent experiments).

Immunostaining for vGluT1, vGAT and VAMP2 showed that vGluT1 positive presynaptic area was significantly increased, whereas vGAT positive presynaptic area correspondingly decreased (Fig. 4a,b,d; no change in intensity of vGluT1 or vGAT immunoreactivity at individual puncta; Fig. 4a,c,e). This indicates that the number of excitatory and inhibitory synaptic terminals is altered upon VPA exposure *in vitro*. *In vivo*, we found PV-positive interneurons to be significantly affected (Fig. 2). These interneurons include fast-spiking basket cells and chandelier cells, which innervate cell soma, proximal dendrites and axon initiation segments^32^. Consistent with an impairment of this class of cells, the number of perisomatic GAD65-positive puncta was severely decreased in VPA-treated cultures (Fig. 4f–i). These data suggest that *in vitro* VPA-exposure during early development decreases the number of GABAergic presynaptic boutons and inversely increases the number of glutamatergic presynaptic boutons, thereby potentially leading to a shift in E/I balance.

**Figure 5:**
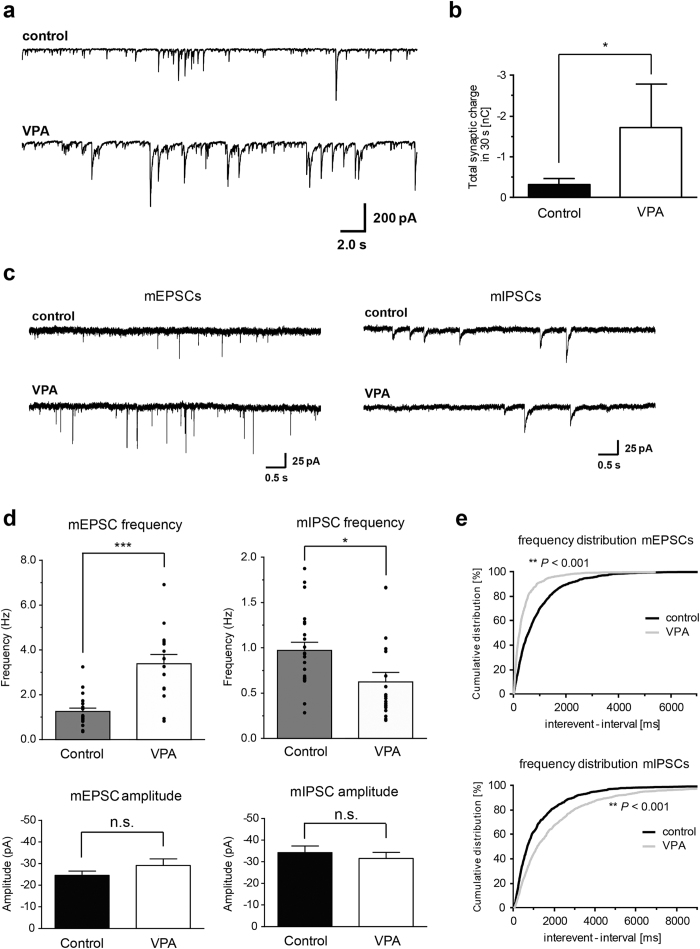
Increased spontaneous synaptic activity and alterations in mEPSC and mIPSC frequency in VPA treated neurons *in-vitro*. (**a,b**) VPA treated neurons show a high frequency of spontaneous synaptic currents with large amplitude indicating increased synaptic network activity compared to untreated neurons (n = 5 cells per condition). (**c**) mEPSCs (left) and mIPSCs (right) were recorded in untreated (upper traces) and VPA treated (lower traces) neurons in the presence of 0.5 μM TTX and 2 μM gabazine or 5 μM CNQX plus 25 μM AP5, respectively. (**d**) In VPA treated neurons (n = 15), the frequency of mEPSCs is significantly increased relative to control (n = 21) whereas the frequency of mIPSCs is significantly decreased (n = 19) as compared to untreated neurons (n = 22). The amplitude of mEPSCs and mIPSCs is not significantly changed between untreated and treated neurons. (**e**) Cumulative probability distribution of interevent interval of mEPSCs (upper panel) and mIPSCs (lower panel) obtained from the same cells as in (**d**).

To directly relate these morphological phenotypes to functional alterations in the neuronal network we performed whole-cell voltage-clamp recordings on VPA-treated and control cultures. We observed a striking increase in spontaneous synaptic network activity after VPA treatment (Fig. 5a,b). To further analyze the functional basis underlying the enhanced network activity, we recorded mEPSCs and mIPSCs in the presence of 0.5 μM TTX. The frequency of mEPSCs was 2.7-fold increased (*P* < 0.001), whereas the mIPSCs frequency was decreased to 64% of control (*P* < 0.05) (Fig. 5c–e). The amplitude of neither mEPSCs nor mIPSCs was significantly affected by VPA treatment (Fig. 5c–e). Similarly, the decay time constant of mEPSCs and mIPSCs was very similar in control and VPA treated cells (mEPSCs: 3.08 ± 0.28 ms, n = 21 versus 3.08+/−0.29 ms, n = 15, P = 0.992 and mIPSCs: 47.0+/−3.7 ms, n = 22 versus 58.6+/−3.3 ms, n = 19, *P* = 0.026), suggesting that neither density nor subunit composition of postsynaptic GABA_A_ and AMPA receptors is substantially affected. In combination with the morphological data obtained for the *in vitro* preparations, these findings demonstrate that *in vitro* VPA treatment during early neural development leads to enhanced network activity generated by an imbalance in the number of excitatory and inhibitory synaptic connections.

### VPA treatment and pharmacological HDAC inhibition produce similar alterations in synaptic protein levels

**Figure 6:**
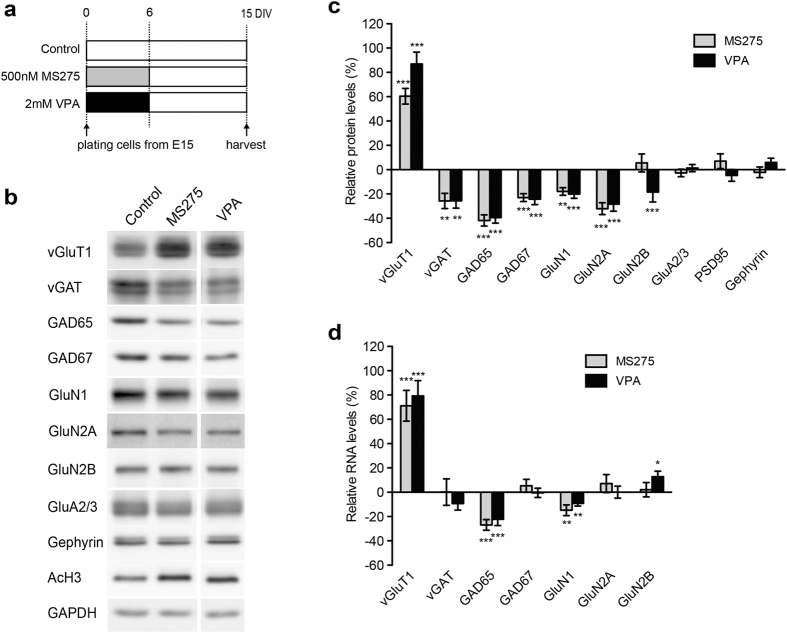
*In vitro* VPA treatment alters expression of GAD65 and vGluT1 through selective HDAC inhibition. (**a**) Scheme of procedure of experiments using a HDAC inhibitor, MS275. (**b,c**) Example western blots and quantification of relative changes in protein level in cultured neocortical neurons treated with MS275 (n = 4–9) or VPA (n = 6–11) as compared to vehicle treated control cultures. Combined blots were run under the same experimental conditions and blots were cropped to display the relevant molecular weight range. Uncropped blots are presented in Fig. S4e. (**d**) Altered mRNA level in cultured cortical neurons treated with MS275 (n = 13) or VPA (n = 11).

VPA is an inhibitor of HDAC1, 2, 3 and 8^33^,^34^,^35^,^36^. To evaluate whether the effect of VPA on synaptic protein levels might rely on HDAC inhibition, we treated cultured neurons with MS275, a selective inhibitor for HDAC1, 2, 3 and 9^33^,^34^,^35^,^36^ (Fig. 6a). MS275 treatment resulted in similar synaptic protein expression profiles as those observed with addition of VPA (Fig. 6b,c). Thus, MS275 reduced vGAT, GAD65 and GAD67 expression, whereas it enhanced vGluT1 expression. Notably, also the transcript levels for GAD65 and vGluT1 mRNAs were significantly altered after VPA and MS275 treatment (Fig. 6d), supporting the notion that these drugs modify presynaptic protein expression through a transcription-based mechanism. Notably, vGAT and GAD67 transcripts were not altered upon VPA or MS275 treatment. Thus, the reduction in vGAT and GAD67 protein levels may represent a secondary effect of a VPA-induced epigenetic change.

**Figure 7:**
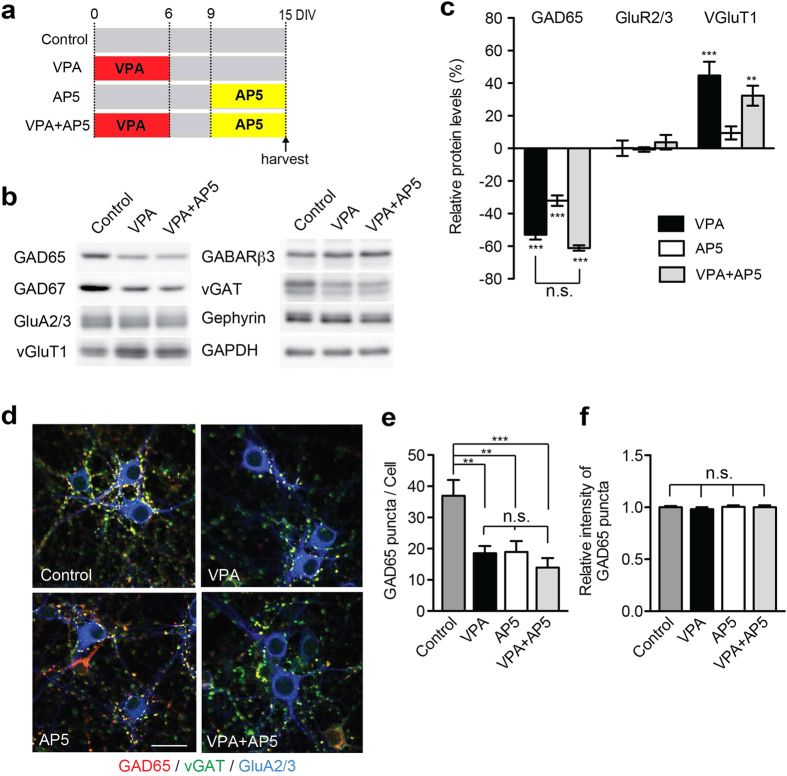
Effect of VPA treatment is occluded by blockade of NMDAR activation in *in vitro* VPA model. (**a**) Scheme of procedure of experiments using the NMDAR antagonist AP5. During the first 6 days of culture, neocortical neurons were exposed to 2 mM VPA or vehicle. Subsequently, AP5 (200 μM) was added to from day-*in-vitro* (DIV) 9–15. (**b,c**) Relative synaptic protein expression levels for neuronal preparations treated as described in panel A. The alteration in GAD65, GAD67 and vGluT1 by VPA is occluded by AP5 treatment (VPA; n = 5–6, AP5; n = 7–13, VPA + AP5; n = 7–9). (**d**) Representative images of endogenous GAD65, vGAT and GluA2/3 protein immunocytochemistry in VPA-, AP5-, or VPA plus AP5-treated cultures. Cultured cortical neurons are triple-stained with anti-GAD65 (red), vGAT (green), and GluA2/3 (blue) antibodies. Scale bar = 20 μm. Blots were cropped to display the relevant molecular weight range. Uncropped blots are presented in Fig. S4f. (**e,f**) Co-application of VPA and AP5 does not have an additive effect on reducing the number of GAD65 puncta. The number of GAD65 perisomatic puncta per cell (**e**) was reduced in VPA, AP5 or VPA + AP5 treated neurons, whereas intensity of GAD65 puncta (**f**) was not altered. 15 or 17 images per condition were analyzed from 3 independent experiments.

HDACs are generally thought to repress transcription. Thus, we hypothesize that the elevation in vGlut1 mRNAs is due to a de-repression resulting from the inhibition of HDAC activities. Considering that GAD65 transcription in interneurons has been linked to activity-dependent neuronal signaling and the transcription factor CREB^37^,^38^, we tested whether GAD65 transcripts in VPA-exposed cortical neurons are modified by NMDAR-dependent neuronal activity manipulation. We probed synaptic protein levels in control and VPA exposed cultured neocortical neurons that were treated with the NMDAR antagonist AP5 for 6 days before harvesting. VPA and AP5 individually resulted in a reduction in basal GAD65 protein level (Fig. 7a–c). Interestingly, effects were not additive when treatments were combined (Fig. 7c). At the morphological level, density of GAD65-positive puncta (but not staining intensity) was reduced upon VPA or AP5 treatments (Fig. 7d–f). Again, combination of both manipulations (VPA and AP5) did not further reduce puncta density as compared to single drug addition (Fig. 7e). These observations provide initial evidence that the reduction in basal GAD65 protein levels observed in VPA-treated cultures may indeed be related to NMDAR-dependent signaling. Finally, the regulated synthesis and release of brain-derived neurotrophic factor (BDNF) represents one potential mechanism of how such NMDAR-dependent signaling may modify GAD65 expression in interneurons^37^,^38^. Using pharmacological manipulations we find that elevation of BDNF transcripts in response to elevated neuronal network activity is somewhat attenuated in VPA-exposed cortical cultures (Fig. S3). These observations are consistent with a role for BDNF in the de-regulation of GAD65 levels in VPA-exposed cells. However, further work will be required to directly test this hypothesis.

In summary, these experiments demonstrate that significant alterations in synaptic components arise at multiple levels. Direct exposure of neurons to VPA or the HDAC inhibitor MS275 elevates expression of some transcripts (including vGluT1). Dysregulation of other transcripts (such as GAD65) is likely a consequence of altered neuronal activity in the VPA-exposed neurons, and other synaptic components are modified only on the protein level.

## Discussion

In this study, we systematically investigated the effect of VPA on synaptic properties with biochemical, cell biological and electrophysiological approaches. We demonstrate a differential impact on glutamatergic and GABAergic components in neocortical neurons, which results in a marked functional E/I imbalance in neuronal networks *in vitro*.

An important conclusion from our experiments is that VPA exposure (either *in utero* or by direct application to cultured neurons) perturbs differentiation in GABAergic interneurons. This perturbation is exhibited by the loss of morphologically recognizable presynaptic terminals in cultured cortical neurons and significant reduction in mIPSC frequency. Thus, there is indeed a loss of functional inhibitory transmission after VPA exposure. In vivo, we observe a similar loss in presynaptic GABAergic markers. Moreover, the reduction in parvalbumin and Kv3.1 protein levels suggest that not only presynaptic markers but also other components are altered in parvalbumin-positive interneurons, a population that encompasses fast-spiking basket and chandelier cells. Whether other interneuron populations are modified remains to be tested experimentally. Our study significantly expands previous work that examined direct exposure of cultured neurons to VPA^19^,^20^,^39^. Importantly, we specifically focused on exposure of embryonic neurons and used VPA concentrations modeled on dose applicable in human patients to more closely mimic the disease-relevant conditions^31^. Finally, the functional data from whole-cell recording provide a critical extension of the data for interpreting their impact on neuronal function. Based on our observations we conclude that VPA exposure of embryonic neurons results in a long-lasting hyperexcitable network state.

In addition to the impairment in inhibitory interneuron maturation we identified a gain in glutamatergic synapse development. Comparison of tissue from adolescent and adult mice indicated a developmental shift in glutamatergic components, in particular the NMDAR complex. While NMDAR subunit expression in the adolescent somatosensory cortex of VPA-exposed mice was reduced it was elevated in the adult tissue. Similarly, in cultured cortical neurons NMDAR subunit expression was reduced upon VPA exposure–consistent with the early developmental stage of the cells isolated from embryonic tissue. The reduction in NMDARs might attenuate activity-dependent expression of several neural genes (including BDNF) which may subsequently alter GABAergic differentiation, maturation or functions in VPA-exposed neurons. However, further studies will be needed to clarify such mechanisms.

We observed a significant up-regulation of the presynaptic vesicle component vGluT1 in cultured neurons, a finding consistent with previous studies using VPA exposure of rats^10^,^19^. This elevation in vGluT1 was accompanied by an increase in mEPSC frequency, confirming that excitatory synapse function is indeed enhanced by *in vitro* VPA treatment. These observations are consistent with a previous study employing genetic inactivation of HDACs in mouse neurons^40^. However, we did not observe a similar elevation in vGluT1 levels in the somatosensory cortices of offspring from VPA-exposed mice. Given that somatosensory neurons receive inputs from several cortical and sub-cortical regions, it is possible that an elevation of glutamatergic synapse function in VPA-exposed animals might be ameliorated by homeostatic regulation during neural development. In fact, a recent study suggests that enhanced NMDAR functions in adolescent VPA mice might be compensated by homeostatic events at later developmental stages^41^. Alternatively, VPA-exposure *in utero* may trigger additional signaling pathways that are not dependent on HDAC inhibition and thereby explain discordance between the observations in the *in vitro* and *in vivo* exposure conditions.

A striking aspect of the VPA model is that a brief prenatal VPA exposure results in persistent alterations in synaptic proteins and synaptic function. *In utero*, VPA may act directly on fetal neurons but may also have indirect effects such as described for prenatal immune challenges^42^. We demonstrate that direct exposure of fetal neurons to VPA *in vitro* replicates several molecular alterations observed in the brains of VPA-exposed mice (Fig. 3). Thus, it is likely that at least some effects of VPA prenatal exposure are due to direct effects on fetal neurons or neuronal precursors rather than additional maternal signaling responses. Considering that another HDAC inhibitor, MS275, recapitulated the altered expression profile (Fig. 4), we propose that prenatal VPA impairs E/I balance in the offspring through long-term epigenetic effects by HDAC inhibition.

Using our experimental approach we detected a significant reduction in GABAergic proteins in the somatosensory cortex of VPA model mice but not the cerebellum, hippocampus and amygdala (Fig. S1a). Clearly, the pathophysiology of autism is not restricted to a single brain area and different aspects of the behavioral phenotype are likely to result from alterations in a large number of specific circuits, including circuits for positive/negative valence, learning-related structures, but also multisensory integration. The differential responsiveness to VPA between neuronal cell-types and brain areas observed in our experiments might be explained by temporal differences in their developmental program between brain areas rather than a selective pharmacological effect.

ASD-like behavioral features can be observed upon VPA exposure during early postnatal period^43^,^44^. Indeed, in this study, developmental aberrations in the mice were evidenced by a delay in eye opening and the maturation of righting reflexes. We speculate that timing and dose of prenatal VPA exposure *in vivo* may be critical factors for the increased risk and severity of offsprings developing ASD.

Finally, from analyzing a large number of litters from VPA-exposed pregnant mice we conclude that the VPA treatment model in mice is subject to significant litter-specific effects (Fig. S1d,e). Thus, great care needs to be taken to use pups from larger numbers of independent litters as experimental replicates, rather than multiple mice from only a small number of litters.

An important conclusion from this study is that neuronal defects arising from prenatal VPA exposure show a significant developmental trajectory. We hypothesize that the prenatal insult results in a long-lasting epigenetic change in some synaptic target genes which then precipitates a number of transcriptional and post-transcriptional alterations–some of them due to altered neuronal activity in the developing circuits. NMDA-receptor subunit expression switches from reduced expression in adolescent mice to elevated expression in the adult. Notably, NMDAR dysfunction may indeed be an important parameter for the pathophysiology as pharmacological manipulation of NMDARs does indeed modify behavioral phenotypes in the VPA rat model^45^,^46^.

Our analysis highlights an early de-regulation of GABAergic proteins and GABAergic transmission in adolescent VPA offspring that is somewhat attenuated in the adult. Dysregulation of GABAergic inhibitory circuits has been implicated in the onset of neurodevelopmental disorders including ASD^47^. GABAergic axons in postmortem brains from ASD patients display a marked decrease in GAD proteins^48^ and defects in GABAergic inhibitory systems have been reported for several rodent ASD models^49^,^50^,^51^. Disruption of GABAergic neurons during early postnatal phase may strongly influence critical period plasticity, an experience-dependent circuit refinement during the early postnatal phase^52^. Therefore, we speculate that disruption of GABAergic neurons (including the PV-positive populations) by *in utero* VPA exposure may represent a primary insult responsible for the neuronal dysfunction in this autism model. These developmental aspects highlight the importance for tailoring treatment approaches and the timing of interventions to maximize their efficacy in this developmental disorder.

## Methods

### Antibodies

Rabbit anti-Neuroligin-1 and anti-Neuroligin-3 were previously described^53^. Rabbit anti-GluR2/3 was a kind gift from Dr.Nathalie Sans. The following commercially available antibodies were used: rabbit anti-vesicular glutamate transporter 1 (vGluT1), rabbit anti-vesicular GABA transporter (vGAT), mouse anti-Vesicle associated membrane protein 2 (VAMP2), rabbit anti-Homer1, mouse anti-gephyrin, mouse anti-NR1 (Synaptic Systems), rabbit anti-GAPDH (Enogene), mouse anti-actin (Sigma), sheep anti-parvalbumin (PV) (R&D), goat anti-calretinin (CR) (Swant), mouse anti-GAD67, anti-NR2A (Upstate Biotechnology), mouse anti-NR2B, mouse anti-PSD95, mouse anti-GABAβ3, mouse anti-Kv3.1b (Neuromab), mouse anti-GAD65 (Developmental Studies Hybridoma Bank), mouse anti-MAP2 (Chemicon), rabbit anti-NeuN (Novus Biologicals), mouse anti-CaMKII (ABR Affinity BioReagents), rabbit anti-acetylhistone3 (Cell Signalling).

Secondary antibodies with minimal interspecies cross-reactivity conjugated to cyanine and Alexa 633, 546 or 488 dyes (Jackson ImmunoResearch and Invitrogen) were used for visualization in immunostaining. Secondary HRP conjugated anti-mouse and anti-rabbit IgG, and IRDye 680 coupled anti-Mouse and IRDye 800 coupled anti-Rabbit IgG for quantitative western blotting were purchased from Jackson and LI-COR Biosciences, respectively.

### VPA Mouse Model

Hsd:ICR (CD-1) mice (Harlan, The Netherlands) were mated, with pregnancy determined by the presence of a vaginal plug on embryonic day 0. Valproic acid oil (VPA, SIGMA) was suspended in 0.9% saline. The dosage was 600 mg/kg, and adjusted according to the body weight of the dam on the day of injection. Treated dams received a single subcutaneous injection on gestational day 13 (embryonic 13 day) and control dams received a single injection of saline. Those dams were housed individually and allowed to raise their own litters. The offspring were used for experiments on postnatal week 3 (postnatal 15–21 days) and 10 (postnatal 70–80 days). All procedures related to animal experimentation were carried out in accordance with approved guidelines and were reviewed and approved by the Kantonales Veterinäramt Basel-Stadt.

### Protein analyses

For reproducibly isolating the somatosensory cortical area, fresh coronal sections from bregma −1.0 to −2.5 mm were taken with Brain Matrix (Mouse, adult, coronal, 1 mm; Zivic Instruments) on ice. The brain tissues or cultured cells were homogenized in lysis buffer (phosphate-buffered saline (PBS), 10 mM Ethylenediaminetetraacetic acid (EDTA), 1% TritonX-100, 0.2% Sodium dodecyl sulfate (SDS) and protease inhibitor cocktail (Roche Applied Science)).

For quantitative western blotting, 15 μg total protein were separated by polyacrylamide gel electrophoresis and transferred on nitrocellulose membrane. For visualization, horseradish peroxidase (HRP)-conjugated secondary antibody and the enhanced chemoluminescent (ECL) detection (Pierce) were used. Signals were acquired using an image analyzer (LAS-3000; Fujifilm). For using Odyssey system (LI-COR Biosciences), blots were blocked in Tris-buffered saline containing 3% Top Block (Lubio Science). Protein expression was determined using IRDye coupled secondary antibodies. Fluorescence images were acquired using the Odyssey System. Signals were quantified with ImageJ software (NIH). Signal intensities were normalized to the internal control GAPDH and/or actin. Blots were cropped to display the relevant molecular weight range.

### RNA analyses

Total RNA was isolated using Trizol reagent (Invitrogen), followed by removal of contaminating DNA using Turbo DNA-free (RNase-free DNase; Ambion). 1 μg of total RNA was reverse transcribed using random hexamers and ImPromII (Promega). Quantitative PCR was performed on a StepOnePlus qPCR system (Applied Biosystems). Custom primer sets (see Table 1) were used with SYBR Green Master Mix (Applied Biosystems) and comparative C_T_ method. The mRNA levels were normalized to that of *Gapdh* mRNA.

### Neuronal cell culture

Dissociated cultures of mouse cortical neurons were prepared from E15 pups (outbred NMRI strain) by dissociation with 0.05% trypsin in presence of DNase I (Roche Applied Science) for 10 min at 37 °C. After cell dissociation, Trypsin (SIGMA) was inactivated by Soybean trypsin inhibitor (Sigma). Cells were plated on 24 well dishes (0.5–1.0 × 10^5^/cm^2^) and maintained in Neurobasal medium (Invitrogen) containing 2% B27 supplement, 2 mM Glutamax and penicillin/streptomycin (Invitrogen) for 15 days. For *in vitro* VPA treatment, 2 mM VPA was added to the culture medium for the first 6 days, medium was removed and cells maintained for another 9 days in normal growth medium.

### Immunohistochemistry, image acquisition and analysis

Cultured neurons were fixed with ice-cold fixative (4% paraformaldehyde in 100 mM phosphate buffer, pH 7.4) for 20 min. After fixation, neurons were permeabilized with the PBS containing 0.1% TritonX-100 for 15 min at room temperature and incubated with blocking solution (5% normal donkey serum in PBS) for at least 30 min at room temperature. Incubation with primary antibodies was for 24 h at 4 °C. For visualization, appropriate secondary antibodies conjugated to Alexa 633, 546 or 488 were used.

Images were captured on a Zeiss LSM5 confocal system (Zeiss) and assembled using Adobe Photoshop and Illustrator Software. Images were analyzed using MetaMorph Software (Molecular Dynamics). A single threshold was set for each staining condition to capture puncta that were clearly distinguishable and to minimize merged structures. For quantification of vGluT1 and vGAT positive puncta, double positive area and intensities at individual puncta with VAMP2 (total presynapse) were measured in randomly selected fields from cultured VPA or control neurons. Three independent experiments were analyzed quantitatively.

Animals were transcardially perfused with fixative (4% paraformaldehyde/15% picric acid in 100 mM phosphate buffer, pH 7.4). Tissues were sectioned at 50 μm in PBS on a vibratome (VT1000S, Leica), floating sections were permeabilized in 10% normal donkey serum and 0.3% TritonX-100 in PBS, and immunostained with the primary antibodies for 24 h at 4 °C. For quantification, images (2.16 mm^2^ per image) wewre acquired from cerebral cortex between layerII/III and layerV on a LSM 5 confocal microscope (Zeiss) and assembled using Adobe Photoshop and Illustrator Software. The number of PV positive cells per field was counted.

### Electrophysiology

For whole-cell voltage-clamp electrophysiological recordings, coverslips with control (untreated) and VPA treated cortical neurons (DIV12 or DIV13) were continuously superfused with ACSF containing 123 mM NaCl, 25 mM D-Glucose, 10 mM HEPES, 25 mM NaHCO_3_, 5 mM KCl, 1 mM NaH_2_PO_4_, 2 mM CaCl_2_ and 1 mM MgCl_2_. Patch pipettes (2.5–4.0 MΩ) were pulled from borosilicate glass tubing with 2 mm outer diameter and 0.5 mm wall thickness (Hilgenberg, Malsfeld, Germany) and filled with internal solution containing 140 mM KCl, 10 mM EGTA, 10 mM HEPES, 10 mM MgCl_2_, 2 mM Na_2_ATP and 0.3 mM NaGTP with a pH of 7.28. All recordings were performed at 22–25 °C. Currents were measured with a Multi Clamp 700B amplifier (Molecular Devices, Palo Alto, CA, USA), filtered at 5 kHz and digitized at 10 kHz using a CED Power1401 interface (Cambridge Electronic Design, Cambridge UK). Data acquisition and analysis were achieved using custom software (FPulse, U. Fröbe, Physiological Institute Freiburg) running under IGOR Pro 6.12 (WaveMetrics, Lake Oswego, OR) and the open source software Stimfit (C. Schmidt-Hieber, University College London, https://code.google.com/p/stimfit/). Spontaneous synaptic activity, miniature excitatory postsynaptic currents (mEPSCs) and miniature inhibitory postsynaptic currents (mIPSCs) were recorded at a membrane potential of −70 mV. Miniature postsynaptic currents were measured in the presence of 0.5 μM TTX and 2 μM gabazine (mEPSCs) or 5 μM CNQX plus 25 μM AP5 (mIPSCs). For detection and analysis of mEPSCs and mIPSCs a two-step template matching algorithm implemented in Stimfit was used, as described previously^54^.

### Statistical analysis

Pairwise comparisons were performed using Student’s t-test or Mann-Whitney Test. For multiple comparisons, analysis of variance (ANOVA) followed by Bonferroni or Dunnett test was used. Data are represented as the mean ± SEM. Cumulative frequency distributions in control and VPA-treated neurons were evaluated using Komolgorov-Smirnov test. Statistical significance is indicated as follows: ***P < 0.001, **P < 0.01, *P < 0.05.

## Additional Information

**How to cite this article**: Iijima, Y. *et al.* Distinct Defects in Synaptic Differentiation of Neocortical Neurons in Response to Prenatal Valproate Exposure. *Sci. Rep.* **6**, 27400; doi: 10.1038/srep27400 (2016).

## Supplementary Material

Supplementary Information

## Figures and Tables

**Table 1:** Oligonucleotide sequences of qPCR primer sets.

Primer (Forward) (Reverse)	Sequence (5′-3′)
vGlut1-F	5**′**- TCA AGG CTC GCC TAA CCA ATT C -3**′**
vGlut1-R	5**′**- TTT CCC TCA GAA ACG CTG GTG -3'
vGAT-F	5**′**- TCC TGG TCA TCG CTT ACT GTC TC-3**′**
vGAT-R	5**′**- CGT CGA TGT AGA ACT TCA CCT TCT C-3'
GAD65-F	5**′**- CCT GGT TAG AGA GGA GGG ACT GA -3**′**
GAD65-R	5**′**- CAT AGT GCT TAT CTT GCT GAA AGA GGT A-3'
GAD67-F	5**′**- GCG GGA GCG GAT CCT AAT A-3**′**
GAD67-R	5**′**- TGG TGC ATC CAT GGG CTA C-3'
GluN1-F	5**′**- CGT GTA TGT CAA GCC CAC AAT GAG-3**′**
GluN1-R	5**′**- CAA CGC AGA AGC CAT AAC AGC AC-3'
GluN2A-F	5**′**- ACA CAG AGC TCA TCC CCA AAG AG-3**′**
GluN2A-R	5**′**- GCA GTG GTT AAG ATC CCA AGA C-3'
GluN2B-F	5**′**- TAG CTA TAG AGG AGC GCC AAT C-3**′**
GluN2B-R	5**′**- ATG TCA TAG ACA GAG GAC TCA CGG -3'
GAPDH-F	5**′**- TGT TCC AGT ATG ACT CCA CTC ACG -3**′**
GAPDH-R	5**′**- AGT AGA CTC CAC GAC ATA CTC AGC -3'
