# Supplementary material for: Distinct Defects in Synaptic Differentiation of Neocortical Neurons in Response to Prenatal Valproate Exposure
Source: Sci Rep. 2016 Jun 6;6:27400. doi: 10.1038/srep27400 (PMC4893673; doi:10.1038/srep27400)
Supplement: Supplementary Information [file srep27400-s1.pdf]

Supplemental Information

Distinct Defects in Synaptic Differentiation of Neocortical Neurons in Response to Prenatal Valproate Exposure

Yoko Iijima, Katharina Behr, Takatoshi Iijima, Barbara Biemans, Josef Bischofberger, Peter Scheiffele

Supplemental Figure legends

Figure S1. Expression profile of synaptic proteins in *in vivo* VPA model

(a) Different effect of *in utero* VPA exposure on expression of synaptic proteins between brain tissues. Relative expression level of several synaptic proteins was compared between three brain tissues, somatosensory cortex (Cx), hippocampus (Hp), cerebellum (Cb) and amygdala (Am) from control and adult VPA-treated mice (n= 5-6 animals per each group). n.t.: not tested

(b) Comparison of relative expression level of several synaptic proteins in somatosensory cortex between three ASD models (VPA-treated, FMR1 KO and Neuroligin-3 R451C knock-in (KI) mice). (n=4 FMR1 KO animals, n=8 NL3 R451C KI animals)

(c) Relative expression level of synaptic proteins in somatosensory cortex of male or female VPA-treated mice in adult (n=24-32 male animals, n=10-11 female animals). Expression level of control is defined as 0.

(d,e) Litter-to-litter variations in VPA offspring. Mean protein levels of GAD65 and GluN2A in offspring are plotted for 12 females treated with VPA during pregnancy (each dot indicates relative protein level in the somatosensory cortex of individual offspring). This data highlights strong litter-dependent effects.

Figure S2. Unchanged overall morphology and number of glutamatergic and GABAergic neurons in neocortical neurons treated with VPA in vitro.

(a) Immunocytochemistry was performed with anti-MAP2 and anti-NeuN antibodies. Scale bar = 50μm.

(b,c) The number of CaMKII-positive glutamatergic or GAD67-positive GABAergic neurons in NeuN-positive total neurons was not altered between VPA-treated and untreated neocortical neurons. Approximately 1500 or 3000 total neurons were analyzed in CaMKII/NeuN or GAD67/NeuN images from 3 independent cultures. Scale bar = 100μm.

Figure S3. Activity-dependent elevation in GAD65 mRNA and protein is dependent on NMDAR activation

(a) Cultured neocortical neurons were treated at day-in-vitro (DIV) 14 with Bicuculline (50 μM) or Bicuculline and AP5 (200 μM) and harvested 6 hrs (RNA analysis) or 24 hrs (protein analysis) after treatment. Relative RNA (qPCR relative to Gapdh) or protein

levels of GAD65 (Western blotting) with or without drug treatment were quantified (n=7 or n=4 from 4 independent experiments, respectively).
(b) VPA-exposure (2 mM during the first 6 days of culture) was combined with treatments with Bic or Bic and AP5 in cortical neurons between DIV14 and 15. Subsequent RNA analysis by qPCR (relative to Gapdh) demonstrates that activity-regulated BDNF expression is attenuated in VPA-exposed neurons. (Bic; n=11 or Bic+AP5; n=3 from 5 or 2 independent experiments, respectively).

Figure S4. Uncropped images of blots presented in Figures 1-7.
(a) Figure 1c. (b) Figure 1e. (c) Figure 2a. (d) Figure 3b. (e) Figure 6b. (f) Figure 7b. Images indicate the membrane used in the western blotting with the indicated antibodies.

Figure S1

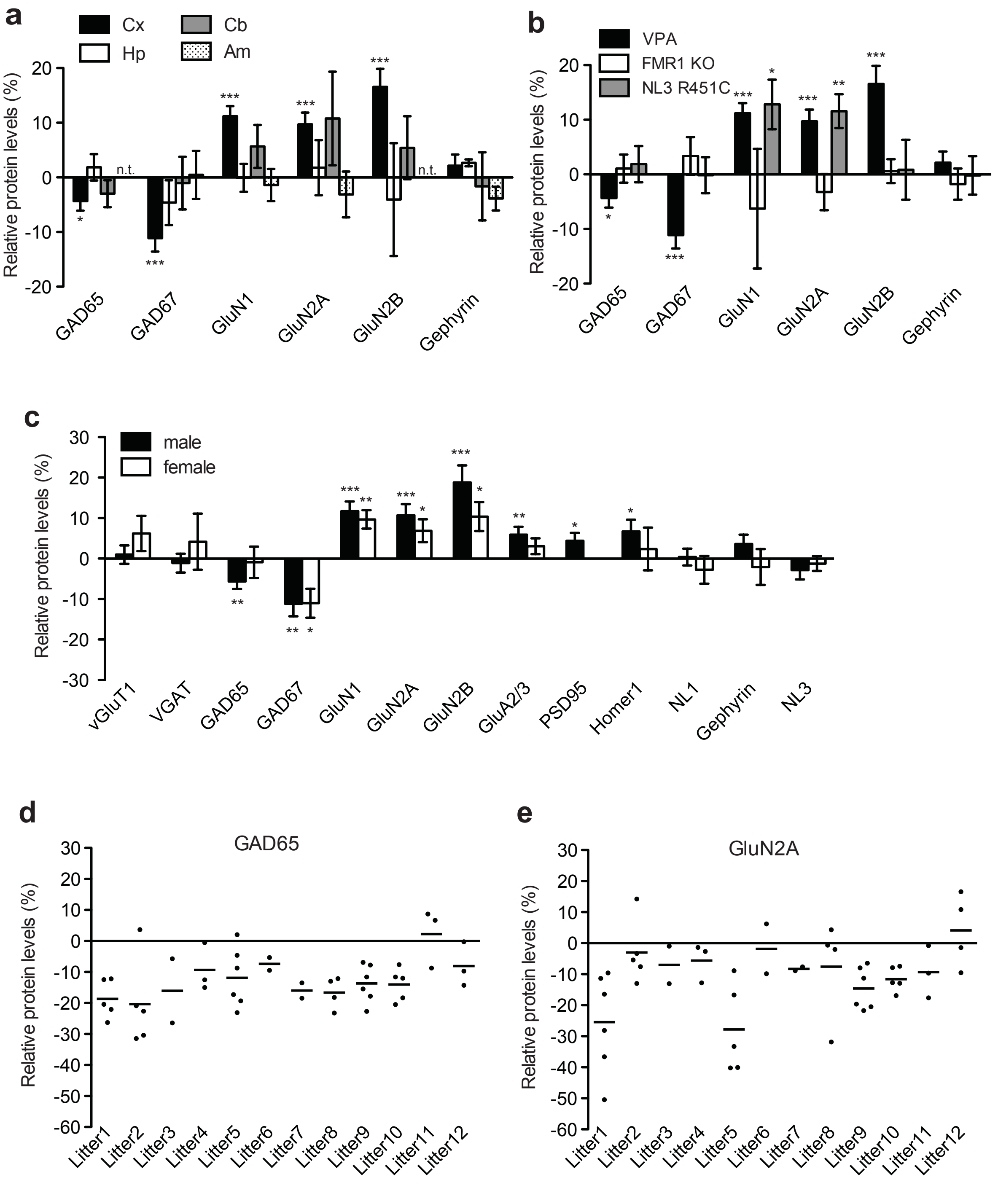
a
Cx
Hp
Cb
Am
Relative protein levels (%)
n.t.
GAD65
GAD67
GluN1
GluN2A
GluN2B
Gephyrin
b
VPA
FMR1 KO
NL3 R451C
Relative protein levels (%)
GAD65
GAD67
GluN1
GluN2A
GluN2B
Gephyrin
c
male
female
Relative protein levels (%)
vGluT1
VGAT
GAD65
GAD67
GluN1
GluN2A
GluN2B
GluA2/3
PSD95
Homer1
NL1
Gephyrin
NL3
d
GAD65
Relative protein levels (%)
Litter1
Litter2
Litter3
Litter4
Litter5
Litter6
Litter7
Litter8
Litter9
Litter10
Litter11
Litter12
e
GluN2A
Relative protein levels (%)
Litter1
Litter2
Litter3
Litter4
Litter5
Litter6
Litter7
Litter8
Litter9
Litter10
Litter11
Litter12

Figure S2

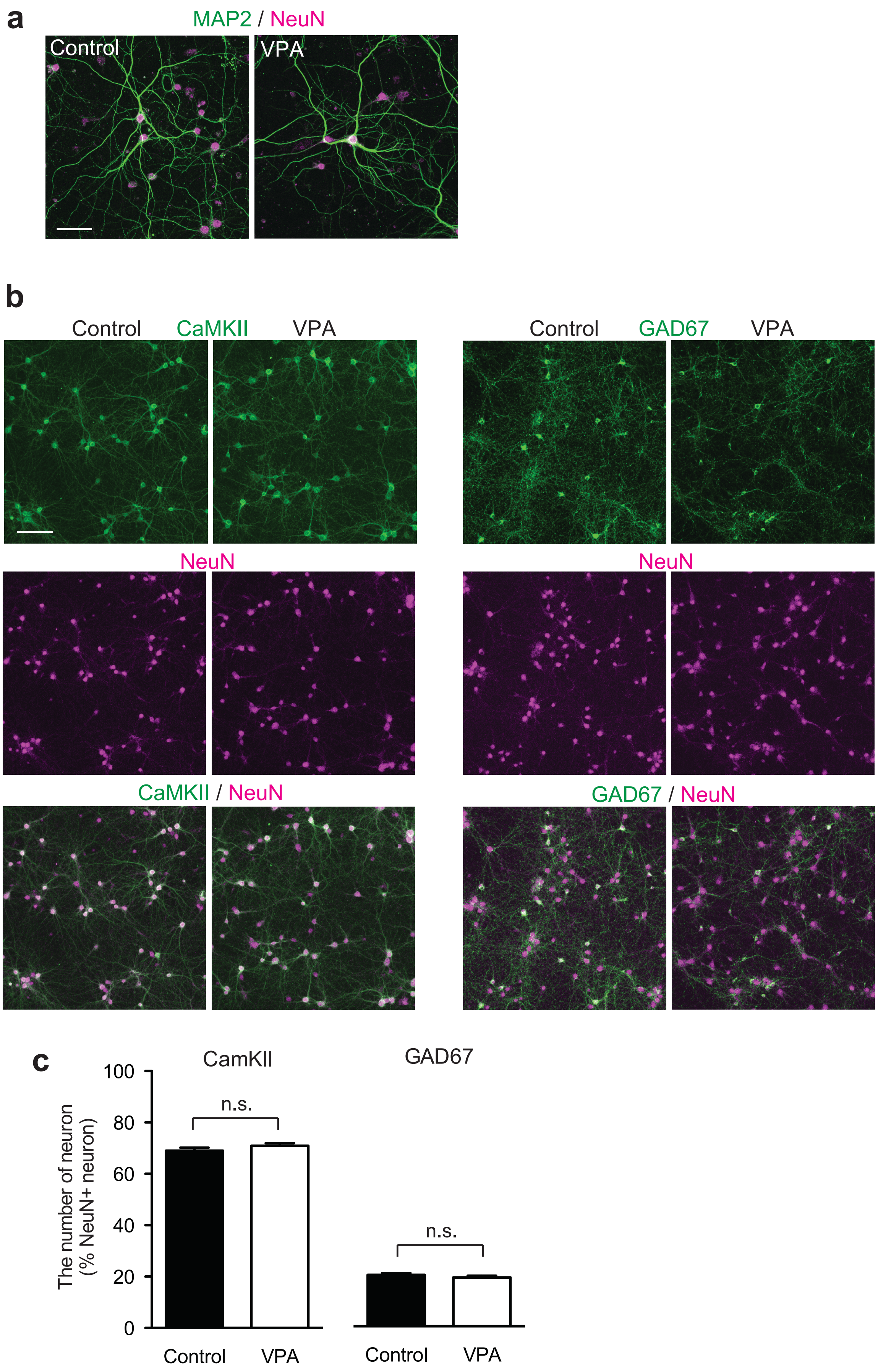
a
MAP2 / NeuN
Control
VPA
b
Control
CaMKII
VPA
Control
GAD67
VPA
NeuN
NeuN
CaMKII / NeuN
GAD67 / NeuN
c
CamKII
GAD67
100
80
60
40
20
0
The number of neuron
(% NeuN+ neuron)
n.s.
n.s.
Control
VPA
Control
VPA

Figure S3

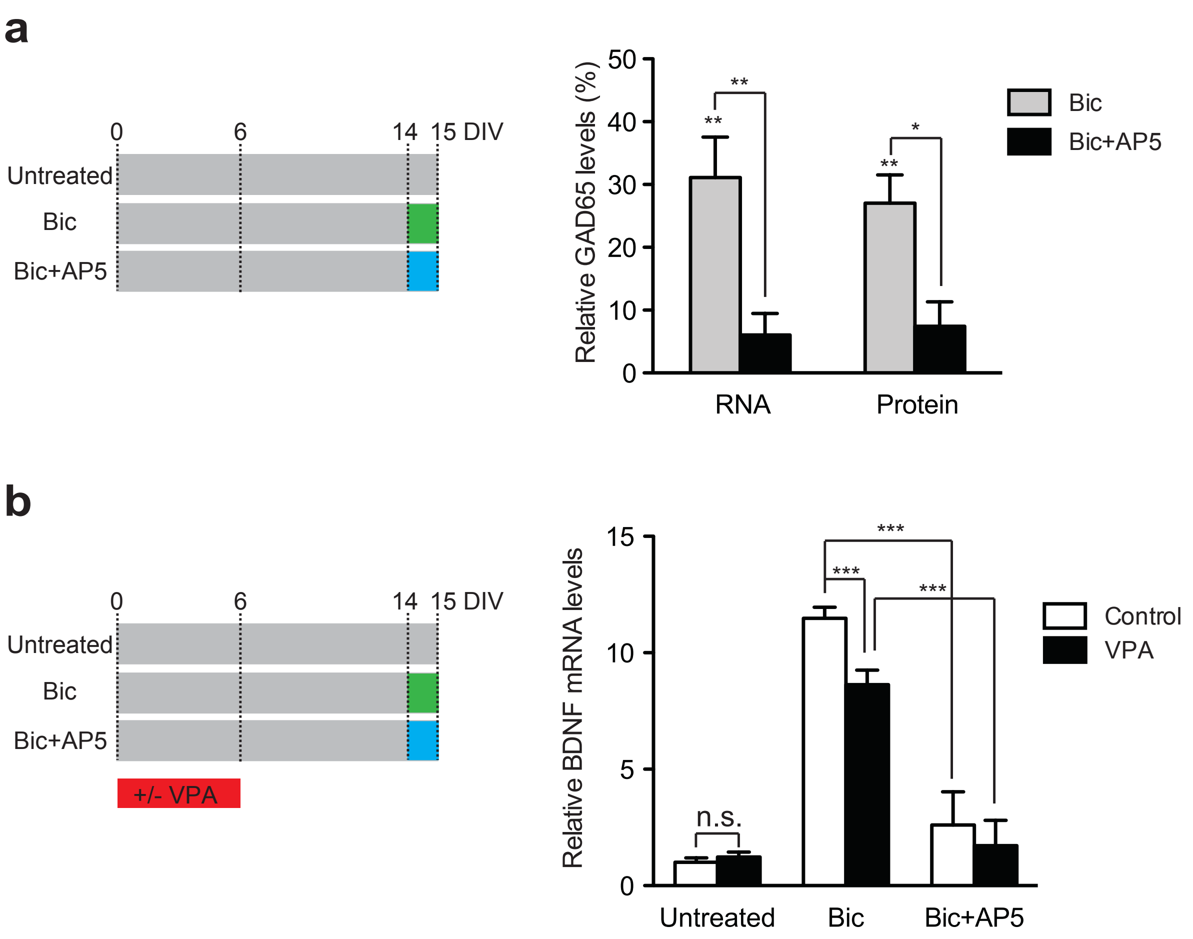
a
0
6
14
15 DIV
Untreated
Bic
Bic+AP5
Relative GAD65 levels (%)
50
40
30
20
10
0
**
**
**
*
RNA
Protein
Bic
Bic+AP5
b
0
6
14
15 DIV
Untreated
Bic
Bic+AP5
+/- VPA
Relative BDNF mRNA levels
15
10
5
0
***
***
***
n.s.
Untreated
Bic
Bic+AP5
Control
VPA

Figure S4

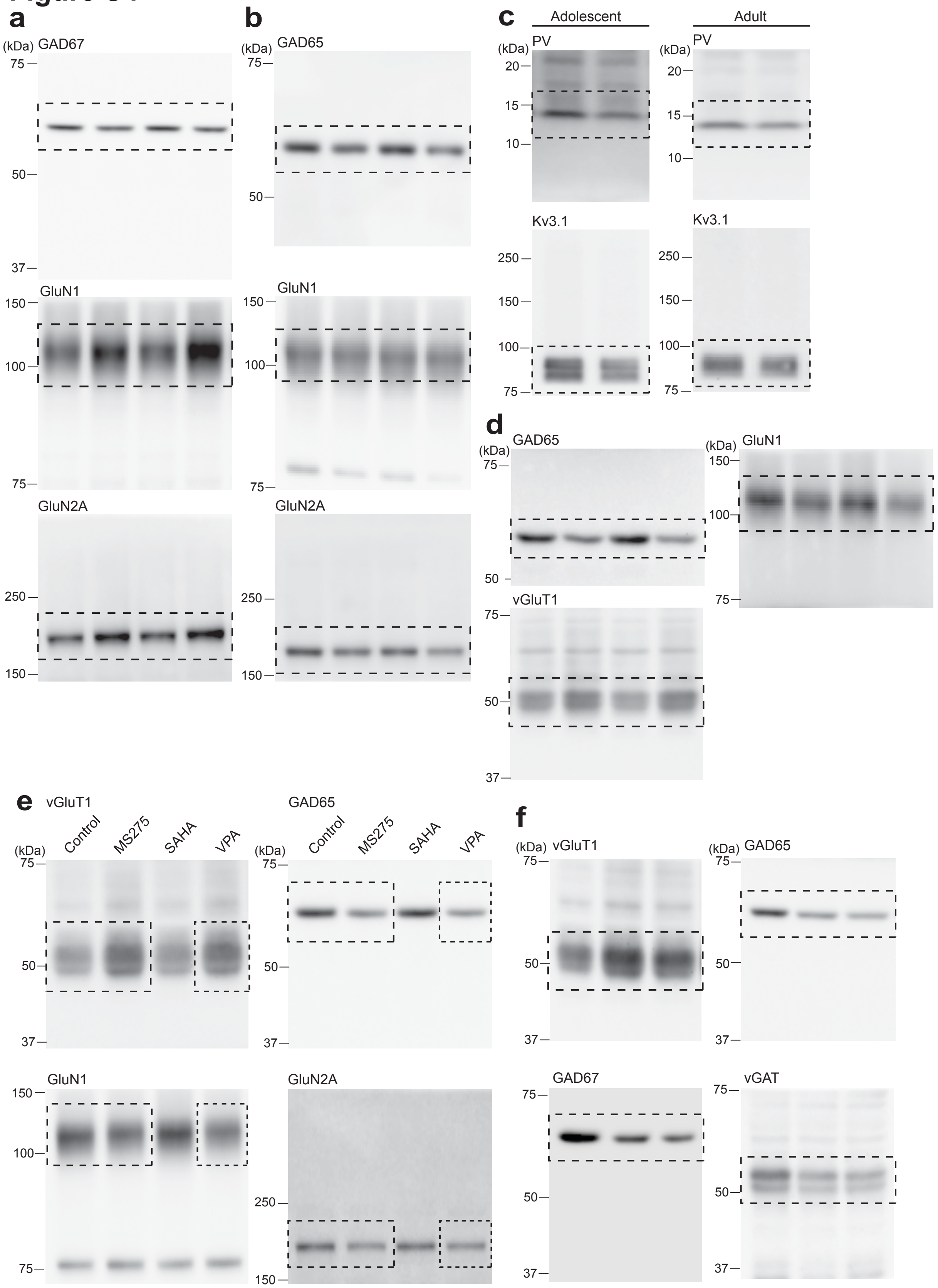

a
b
c
d
e
f
(kDa)
GAD67
GAD65
GluN1
GluN2A
Adolescent
Adult
PV
Kv3.1
vGluT1
vGAT
Control
MS275
SAHA
VPA
250
150
100
75
50
37
20
15
10
